# Neural dynamics of semantic composition

**DOI:** 10.1073/pnas.1903402116

**Published:** 2019-09-30

**Authors:** Bingjiang Lyu, Hun S. Choi, William D. Marslen-Wilson, Alex Clarke, Billi Randall, Lorraine K. Tyler

**Affiliations:** ^a^Centre for Speech, Language and the Brain, Department of Psychology, University of Cambridge, CB2 3EB Cambridge, United Kingdom

**Keywords:** speech, EEG/MEG, computational modelling, RSA, directed connectivity

## Abstract

Human speech comprehension is remarkable for its immediacy and rapidity. The listener interprets an incrementally delivered auditory input, millisecond by millisecond as it is heard, in terms of complex multilevel representations of relevant linguistic and nonlinguistic knowledge. Central to this process are the neural computations involved in semantic combination, whereby the meanings of words are combined into more complex representations, as in the combination of a verb and its following direct object (DO) noun (e.g., “eat the apple”). These combinatorial processes form the backbone for incremental interpretation, enabling listeners to integrate the meaning of each word as it is heard into their dynamic interpretation of the current utterance. Focusing on the verb-DO noun relationship in simple spoken sentences, we applied multivariate pattern analysis and computational semantic modeling to source-localized electro/magnetoencephalographic data to map out the specific representational constraints that are constructed as each word is heard, and to determine how these constraints guide the interpretation of subsequent words in the utterance. Comparing context-independent semantic models of the DO noun with contextually constrained noun models reflecting the semantic properties of the preceding verb, we found that only the contextually constrained model showed a significant fit to the brain data. Pattern-based measures of directed connectivity across the left hemisphere language network revealed a continuous information flow among temporal, inferior frontal, and inferior parietal regions, underpinning the verb’s modification of the DO noun’s activated semantics. These results provide a plausible neural substrate for seamless real-time incremental interpretation on the observed millisecond time scales.

## Significance

The rapid comprehension of speech is a remarkable but poorly understood human capacity. Central to this process is the integration of the meaning of each word, as it is heard, into the listener’s interpretation of the utterance. Here we focus on the real-time flow of neural activity that underpins this combinatorial process, using multivariate pattern analysis and computational semantic models to discover the contextual constraints that are constructed as each word is heard, and to determine how these constraints guide the interpretation of future words in the utterance. This combination of methods reveals a continuous information flow across the left-hemisphere language system, strongly constraining the immediate activation of word meanings and providing a neural substrate for seamless real-time speech comprehension.

Understanding spoken language involves an extensive and complex set of neural computations. Central to these are the processes involved in semantic composition, whereby the meanings of words are combined into more complex representations, such as the combination of a modifier and noun (e.g., “green dress”) or, as in the current study, a verb and its direct object (DO) noun (e.g., “eat the apple”). These combinatorial processes form the backbone of the incremental interpretation of spoken language, enabling listeners to integrate the meaning of each word as it is heard into a dynamically modulated multilevel representation of the preceding words of the utterance.

There has been a long-standing, broad-based interest in semantic combination, initially involving behavioral studies of how contextual constraints affect semantic access (1) and semantic flexibility (2) and more recently focusing on the neural substrates for these processes. In this more recent literature, the combination of word meanings has principally been discussed either as a process of integration or unification involving interactions between the left inferior frontal gyrus (LIFG) and left posterior middle temporal gyrus (LpMTG) (3–6) or as a syntactically licensed combination of individual word meanings involving primarily the left anterior temporal lobe (LATL) (7, 8). Recent neuroimaging studies have also identified the left angular gyrus (LAG) (9–11) as well as the LATL (12, 13) as regions involved in semantic combination, with a recent magnetoencephalographic (MEG) study showing that LATL activity precedes activity in the frontal cortex during combinatory semantic processing (13). However, while this research provides an overall picture of the brain regions underpinning semantic combination, relatively little is known about the specific neural dynamics of these processes, or about the combinatorial mechanisms by which the meaning of each word is selectively integrated into its utterance context. Historically, most studies have either used poorly time-resolved functional magnetic resonance imaging (fMRI) methods or depended on event-related potential measures (most saliently the N400) that are spatiotemporally diffuse and not in themselves fully understood. Many studies, moreover, depend on relatively blunt contrasts of phrases or sentences against lists of words or pseudowords that cannot be combined (10, 12–17) and have not directly modeled the semantics of the individual words tested and have not been able to measure the precise timing of the specific processes involved.

Building on the important but incomplete picture provided by earlier research, the present study combines real-time neuroimaging measurements with recent developments in multivariate statistics and computational linguistics to probe directly the specific neurocomputational content of what is being computed during incremental semantic combination and to determine where and when in the brain these computations take place. We used topic modeling, a corpus-based computational linguistic method that has been widely used in machine learning and natural language processing (18), to build explicit, quantifiable models of the semantics of successive words, focusing here on the integration of the semantics of a verb and its DO noun in verb-DO noun sequences (e.g., “ate the apple”) placed in short contexts, such as “the elderly man ate the apple.” The topic modeling method makes it possible to specify the context-independent semantics of each DO noun, and to test how the specific semantic constraints provided by the preceding verb interact with the activation of DO noun semantics millisecond by millisecond as the noun is heard. Critically, using these probabilistic semantic models, we used spatiotemporal searchlight representational similarity analysis (ssRSA) (19, 20), operating in electro/magnetoencephalographic (EMEG) source space, to compare the similarity structure of contrasting models of DO noun semantics with the similarity structure of observed patterns of brain activity, making it possible to determine which specific semantic contents of the DO noun are encoded across the brain over time. We also used a novel measure of dynamic directed connectivity to probe the precise timing and the directionality of information flow between critical brain regions (21, 22). Whole-brain EMEG data were collected as participants listened naturally to these sequences (with no overt task) and was source-localized for all the analyses reported here.

This combination of methods not only provides uniquely detailed access to the neural infrastructure for human language comprehension in general, but also enables us to address the long-standing but still controversial issue of how and whether word meanings are flexibly interpreted in the context in which they occur (23–28) or whether they have context-independent properties that are always present in the neural instantiation of the meaning of a word (29, 30). Previous psycholinguistic studies have shown that a word’s meaning is flexibly interpreted in the context in which it occurs (23, 24) with, in the strongest case, only the contextually relevant meaning of the word being activated (25–28). We test this hypothesis for contextualized semantic representation using topic modeling to transparently represent the semantic contents of each successive word and to determine how and when these contents change as a function of dynamic neurally represented contextual constraints.

In the next section, we present the progression of integrated, interdependent analyses using a range of different methods, that are necessary to construct and validate an account of the detailed neurocomputational underpinnings of dynamic semantic combination in a spoken sentential context. The starting point is the construction of quantifiable semantic models of the specific semantic properties of each verb and each DO noun, using the topic modeling approach. The neurocomputational goodness of fit of these models is then tested against EMEG brain data using ssRSA, for a set of models of verb semantics. Following the demonstration of significant verb semantic model fit, we focus on verb–DO noun interaction, comparing the brain data model fit of content-independent models of DO noun semantics against contextualized DO noun models that reflect verb semantic constraints. Given the strong constraint effects observed in these comparisons, we then go on to investigate the neuroanatomical locations of the interactions between verb semantic constraints and DO noun semantics and, finally, to establish the timing and directionality of neural information flow between these critical regions.

## Results

### Topic Modeling for Verb and DO Noun Semantics.

To probe the neural mechanisms underpinning how verb semantic constraints are generated and used to constrain the semantic interpretation of the upcoming DO noun, we constructed sets of 6 spoken sentences of the form “subject noun phrase (SNP) + verb + DO noun” (e.g., “The elderly man ate the apple”). To generate a broad range of variation in degree of constraint between the verb and the DO noun, 3 different verbs were selected for each sentence set, with each verb being paired with 2 different DO nouns. Sixty sets of this type were constructed, giving a total of 360 sentences (*Methods*). For each DO noun, the 3 preceding verbs varied in both the content and strength of the semantic constraints they placed on it. For example, “eat” constrains its DO noun toward something edible, “hold” is more likely to be followed by objects that are small or light, while “want” has less specific preferences over a following DO noun.

**Fig. 1.**
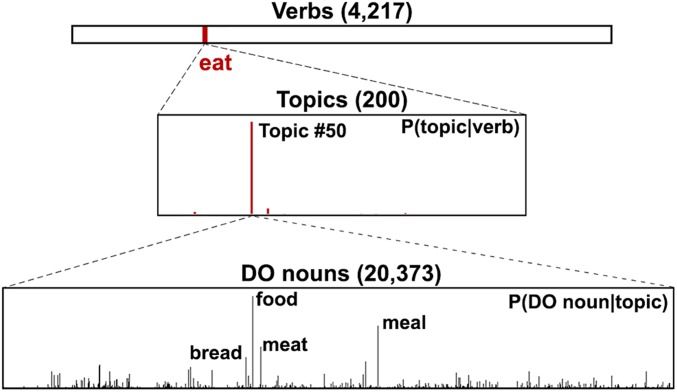
Example of topic modeling results. Each verb (*Upper*) (e.g., “eat”) is represented as a distribution over 200 semantic topics (*Middle*), P(topic|verb), which reflects its semantic constraints over the DO noun. Each topic is a distribution over the vocabulary consisting of all the DO nouns from the large-scale corpora (*Lower*), P(DO noun|topic). Moreover, the meaning of a topic can be readily interpreted by the top words ranked by probability (e.g., topic 50 is a food topic).

**Fig. 2.**
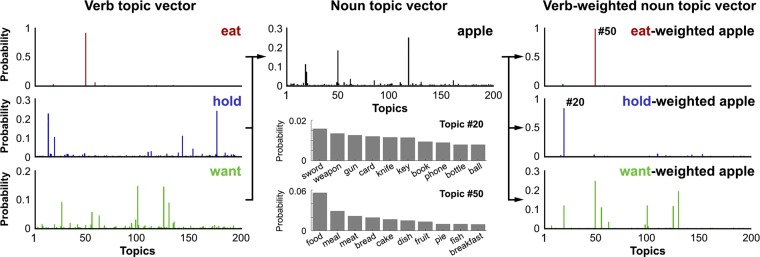
Examples of verb (*Left*) and noun (*Middle*) topic vectors that separately capture verb semantic constraints and DO noun semantics. The verb-weighted noun topic vector (*Right*) models the meaning of the DO noun in the context of a prior verb by emphasizing topics that are preferred by the preceding verb through element-by-element multiplication between verb and noun topic vectors.

To model the semantics of the verbs and the DO nouns, we adopted the topic modeling method known as latent Dirichlet allocation (LDA) (31). This is a generative probabilistic approach aimed at extracting the latent semantic topics from large-scale corpora. Using the co-occurrence frequency between verb and DO noun as training data (32), LDA resulted in 200 topics (*Methods* and *SI Appendix*, section 4), with each topic a probabilistic distribution over the whole vocabulary of DO nouns from the large-scale corpora included in model training. Importantly, the meaning of a topic can be inferred from the highest-ranking words in terms of their probability, that is, P(DO noun|topic). For example, if a topic prefers words like, “meal,” “meat,” “cake,” and “bread,” then it could be plausibly labeled a “food” topic (Fig. 1, *Lower*). Each verb can be represented as a verb topic vector which quantifies its semantic constraints on the following DO noun as a unique distribution over the 200 topics, that is, P(topic|verb) (Fig. 1, *Middle*). Similarly, a noun topic vector can be obtained to model the semantics of a DO noun (*Methods*), which is also a distribution over the same 200 topics, that is, P(topic|DO noun). In this way, we quantified verb and noun semantics separately using vectors in the semantic space constructed by the 200 latent semantic topics (Fig. 2, *Left* and *Middle*).

**Fig. 3.**
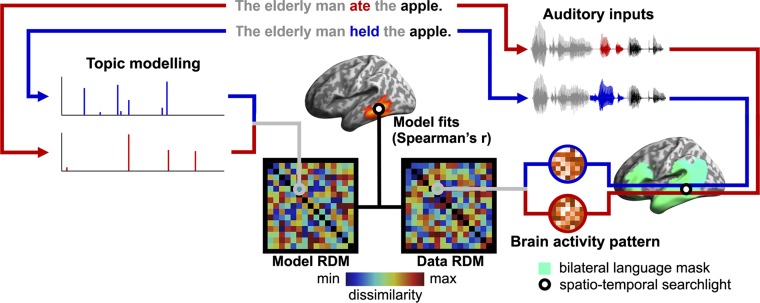
Illustration of the pipeline for ssRSA that correlates the dissimilarity generated by topic modeling (i.e., model RDM) and that encoded by brain activity (i.e., data RDM) using a spatiotemporal searchlight moving within a bilateral language mask at each time point during speech input. Model fits reflect when and where the information captured by the model is represented in the brain.

Within the framework of ssRSA, these verb and noun topic vectors were then used to construct a series of model representational dissimilarity matrices (model RDMs), which were correlated with data RDMs extracted from source-localized EMEG data within a spatial-temporal searchlight moving across a bilateral language mask (33–35) (Fig. 3 and *Methods*). This enabled us to assess the neurocomputational goodness of fit of these distributional semantic models and thereby determine whether, when, and where the information captured by these computational models is encoded in the brain.

### Neural Model Fit for Verb Semantic Models.

**Fig. 4.**
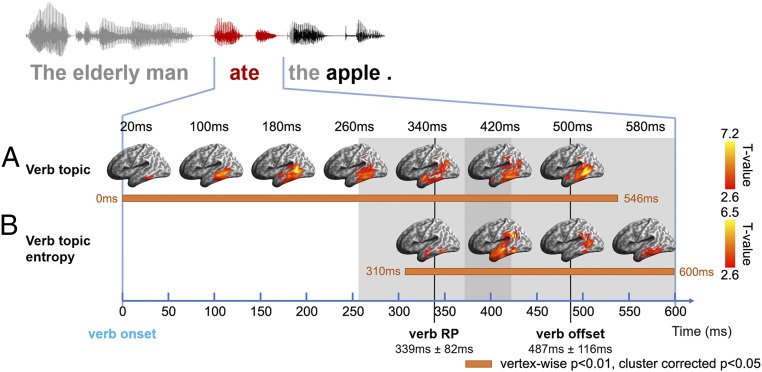
ssRSA results of model RDMs during the verb epoch (aligned to verb onset, extending 600 ms afterward to cover 1 SD of verb duration). (*A*) Verb topic RDM that captures verb semantics. (*B*) Verb topic entropy RDM modeling the strength of a verb’s semantic constraints. Significance was determined by 5,000 nonparametric permutations with vertex-wise *P* < 0.01 and cluster-wise *P* < 0.05. Horizontal orange bars indicate periods during which different model RDMs showed significant effects. Gray shading indicates the range of 1 SD for verb RP and verb offset.

Testing initially for the neural distribution of verb semantic constraints, as defined by topic modeling, we constructed a verb topic model RDM based on the cosine distance between the topic vectors of verbs in different sentences. The verb topic RDM was tested against source-localized EMEG data RDMs within an epoch aligned to verb onset and extending 600 ms forward from this point (verb duration 487 ± 116 ms). The mean recognition point (RP) of the verb—the point in the speech input at which it differentiates from other cohort candidates and can be uniquely identified (36)—was 339 ± 82 ms after verb onset as estimated using CELEX (37) (Fig. 4). During this epoch, we found significant model fit (i.e., Spearman’s rank correlation between model RDM and data RDM) for the verb topic RDM in the LpMTG. Weak model fit can be seen already at verb onset, with stronger effects emerging within 50 to 100 ms after onset and peaking close to verb RP in LpMTG. The effects extended anteriorly into the LATL as verb RP approached and spread posteriorly into the left supramarginal gyrus (SMG) and angular gyrus (AG) and persisted until verb offset (vertex-wise *P* < 0.01, cluster-wise corrected *P* < 0.05 with 5,000 nonparametric permutations, as applied to all reported ssRSA results; ref. 38) (Fig. 4*A*). Note that the verb topic effects detectable at verb onset are likely to reflect the shared properties of the subject noun and verb (*SI Appendix*, Fig. S2), which are already activated as soon as the subject noun is recognized (*SI Appendix*, section 1). Critically, however, for the purposes of the current study, these further analyses show that only verb-specific model fit is seen after verb RP, continuing until verb offset (*SI Appendix*, Fig. S3).

The verb topic vector provides information about both the content (i.e., what topics a verb constrains toward) and the strength of semantic constraints (i.e., the shape of the distribution over topics, with a more focused distribution indicating higher constraint strength and lower uncertainty). Although these 2 aspects together determine a verb’s semantic constraints, we can separate out the strength of constraint by calculating the entropy embedded in verb topic vectors (*Methods*). A verb exhibits high constraint strength by showing preferences for only a few topics (i.e., low entropy), which results in less uncertainty about the likely properties of the following DO noun and vice versa for low constraint verbs. The wide range of the strength of semantic constraint across the verbs used in this study is captured by the distribution of verb topic entropy (*SI Appendix*, Fig. S4). We constructed the verb topic entropy RDM by taking the absolute difference between the entropy of each verb topic vector. Significant model fit for this model RDM, exhibiting sensitivity to constraint strength, emerged much later than for the verb topic RDM, first appearing in the left middle temporal gyrus (LMTG) at 310 ms from verb onset, around verb RP, and then extending briefly into the LATL and L SMG/AG before focusing around LpMTG toward verb offset (Figs. 4*B* and 6*B*).

### Verb Semantic Constraints and the Activation of Noun Meaning.

In the context of these results for models of verb semantic constraints, we can then ask how these constraints interact with the access and interpretation of the following DO noun. Are only the subset of noun semantics preferred by the verb significantly activated when listeners hear the DO noun? Or are the initially activated semantics of the noun unaffected by verb constraints, providing evidence for exhaustive access to its context-independent semantics? To model the potential effects of a verb’s semantic constraints on its DO noun, we constructed verb-weighted noun topic vectors through element-by-element multiplication between the verb topic and noun topic vectors. This results in a verb-weighted noun topic vector that contains only topics preferred by both the verb and its DO noun (Fig. 2, *Right*). Since each topic is a probabilistic distribution over the vocabulary of DO nouns from the large-scale corpora, the verb topic vector reflects the semantic constraints of a verb, that is, what a verb “expects.” In contrast, the noun topic vector models the semantic contents of a DO noun by specifying what it potentially “offers.” Thus, although the multiplication between topic vectors is a symmetrical manipulation, the verb-weighted noun topic vector depicts the DO noun’s semantic representation in the directional context of the prior verb’s semantic constraints. The resulting verb-weighted topic RDM and noun topic RDM capture verb-constrained noun semantics and context-independent noun semantics, respectively. These model RDMs were obtained by calculating the cosine distances between the corresponding topic vectors. Note that the noun topic RDM is considered to capture context-independent semantics because the topic modeling included every occurrence of a DO noun across very large corpora, resulting in DO noun semantic representations not biased by any specific context.

**Fig. 5.**
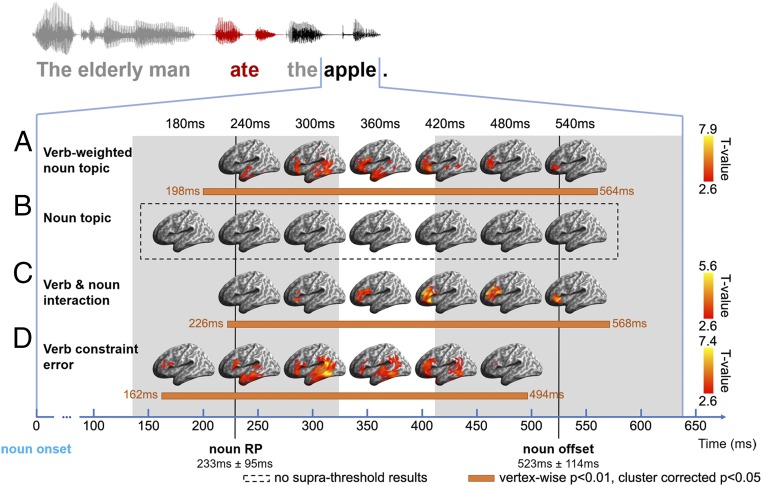
ssRSA results of model RDMs during the noun epoch (aligned to noun onset, extending forward by 640 ms to cover 1 SD of noun duration). (*A*) Verb-weighted noun topic RDM that captures noun semantics as modified by the prior verb. (*B*) Noun topic RDM that models the context-independent semantics of the DO noun. (*C*) Verb–noun interaction RDM reflecting the interaction between verb and noun semantics. (*D*) Verb constraint error RDM measuring the ease with which the DO noun fits into the semantic constraints placed by the prior verb. Significance was determined by 5,000 nonparametric permutations with vertex-wise *P* < 0.01 and cluster-wise *P* < 0.05. Horizontal orange bars indicate significant periods for different model RDMs. The gray shading indicates the range of 1 SD for noun RP and noun offset.

**Fig. 6.**
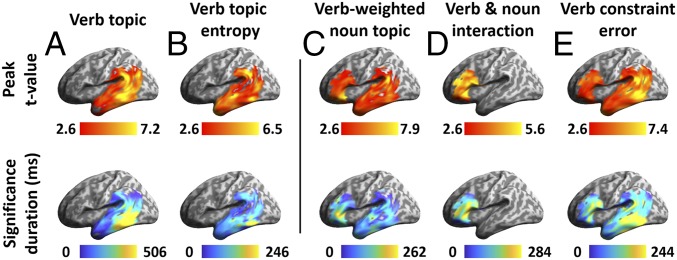
Vertex-wise peak *t* value and significance duration of model RDMs during the verb epoch—verb topic RDM (*A*) and verb topic entropy RDM (*B*)—and during the noun epoch—verb-weight noun topic RDM (*C*), verb–noun interaction RDM (*D*), and verb constraint error RDM (*E*).

We generated an epoch aligned to DO noun onset, 640 ms long (mean DO noun duration 523 ± 114 ms). The RP of these DO nouns in their sentential contexts was on average 233 ± 95 ms from noun onset, as estimated by a behavioral gating test (39, 40) (*Methods*). The verb-weighted noun topic RDM showed significant effects in both left temporal regions and the LIFG concurrent with identification of the noun (around noun RP), starting from 198 ms and 244 ms after noun onset, respectively (Fig. 5*A*). The temporal lobe effects first emerged anteriorly, in the LATL, and then propagated to posterior temporal regions with stronger model fit, finally ceasing before noun offset (Figs. 5*A* and 6*C*). Effects in the LIFG began slightly after those in the temporal cortex and peaked in BA47 after noun RP, lasting until noun offset. In striking contrast, the context-independent noun topic RDM showed no significant effects at any point across this epoch (Fig. 5*B*). Taken together, these results strongly support the hypothesis that only the subset of a word’s semantics constrained by the current sentential context is initially activated (25–28).

### Interactions between Verb Semantic Constraints and Noun Semantics.

To investigate the neural substrates subserving the strong interaction that we observed between verb semantic constraints and noun semantics, we partialled out both the verb topic RDM and the noun topic RDM from the verb-weighted noun topic RDM. Any remaining model fit across the DO noun epoch can be attributed to the interaction between the verb and its DO noun. We found significant effects primarily in left BA45 around noun RP, followed by later model fit in left BA47 (Figs. 5*C* and 6*D*). In contrast to the verb-weighted noun effects, which peaked in left BA47 (Fig. 6*C*), the relatively stronger effects in BA45 for the verb–DO noun interaction may reflect the different roles of these LIFG subdivisions (41–43).

We also constructed a verb constraint error RDM to quantify the processing load involved during the process of semantic integration in fitting DO noun semantics to the constraints placed by the prior verb. Verb constraint error was defined as the cosine distance between the verb topic and noun topic vectors. The greater the overlap between a verb’s semantic constraints and the following DO noun’s semantics, the smaller the distance between the corresponding topic vectors, as reflected by a lower constraint error. Significant effects of this model RDM initially appeared in the left BA45 and LATL around DO noun RP and then extended into more posterior temporal regions as well as the L SMG/AG, peaking in the LpMTG after the DO noun was identified (Figs. 5*D* and 6*E*).

### Mechanisms of Combination: Temporal Patterns of Information Flow between Active Brain Regions.

**Fig. 7.**
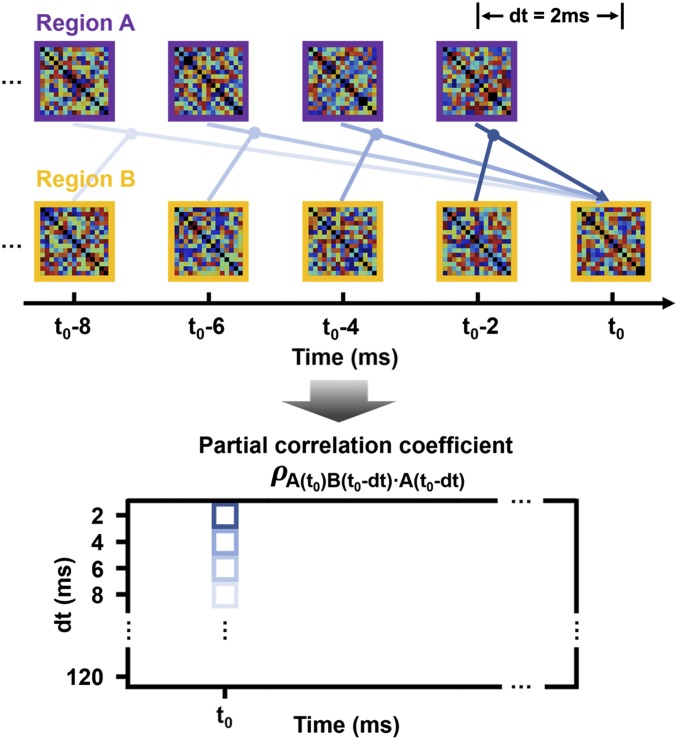
Directed connectivity analysis based on data RDMs constructed separately from two brain regions. (*Left*) The logic is that if region A has causal effects on region B, then the activity of A at a previous time point can be used to explain the current activity in B better than using only the previous activity of B alone, which is quantified by the partial correlation coefficients. (*Right*) The horizontal axis indicates the real time at which the speech unfolds, and the vertical axis indicates the time interval between the current time point and the previous time point used to calculate directed connectivity, thereby providing additional temporal information about the onset and duration of directed connectivity. *t*_0_, current time point; *dt*, time interval between current and previous time points.

To understand the neural mechanisms underpinning how different brain regions cooperate to generate semantic constraints during the meaning composition of adjacent words, we adopted a data-driven method to estimate the information flow between brain regions using their data RDMs (21, 22). The underlying logic here is the same as that of Granger causality analysis (GCA) (44)—that is, if region A has causal effects on region B, then the current activity of B is better explained by taking the previous activity of A into account rather than only using the previous activity of B itself. We quantified the directed connectivity from A to B as the partial correlation coefficient between the activity of A at a previous time point and the current activity of B (as captured by their data RDMs), partialling out the previous activity of B itself (Fig. 7, *Upper*). To avoid possible bias due to the choice of any specific previous time point, we calculated directed connectivity based on a series of time points ranging from 2 ms to 120 ms before the current time point (*Methods*). Based on this extended temporal dimension (i.e., dt in Fig. 7, *Lower*), we can determine the extent to which the current activity in the target region is correlated with the source region’s activity at each time point within the previous 120 ms, which can be used to further infer the delay and duration of potential directed connectivity effects. This method differs from traditional GCA by providing a highly time-resolved profile for the temporal dynamics of information flow between brain regions, adding additional precision to the investigation of the neural dynamics underpinning incremental speech interpretation.

**Fig. 8.**
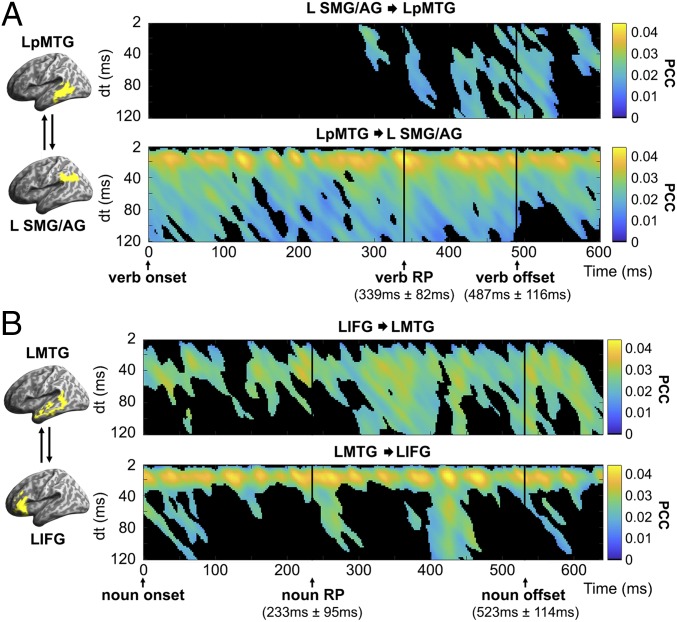
Directed connectivity results for (*A*) the L SMG/AG and LpMTG, showing significant model fit to the verb topic RDM during the verb epoch, and (*B*) the LIFG and LMTG, exhibiting significant model fit to the verb-weighted noun topic RDM during the noun epoch. Significance was determined by 5,000 nonparametric permutations with time point-wise *P* < 0.001 and cluster-wise *P* < 0.01. *dt*, time interval between the current time point and the previous time-point used to calculate directed connectivity; PCC, partial correlation coefficient.

Looking first at the verb epoch, the most significant model fit for the verb topic RDM was found in the LpMTG and L SMG/AG (Fig. 6*A*). On the assumption that the simultaneous model fit in these 2 areas reflects likely information flow between them, we examined the potential directed connectivity between these 2 regions. Prominent effects of directed connectivity from the LpMTG to L SMG/AG were consistently apparent, with a dt value of ∼20 ms (Fig. 8 *A*, *Lower*), indicating that the current activity in the L SMG/AG was significantly correlated with the activity in the LpMTG 20 ms earlier. This suggests that information originating in the LpMTG was constantly delivered to the L SMG/AG with a delay of ∼20 ms as the verb unfolded over time. In contrast, the inferred information flow from the L SMG/AG to LpMTG could be detected only after the verb RP (Fig. 8 *A*, *Upper*).

Turning to the DO noun epoch (Fig. 8*B*), we calculated the directed connectivity for the regions in the LMTG and LIFG that showed the most significant model fits for the verb-weighted noun topic RDM (Fig. 6*C*). Information flow from the LMTG to LIFG showed a similar temporal pattern to the relationship between the LpMTG and L SMG/AG in the verb epoch, with a continuous correlational relationship rapidly updated at delays of ∼20 ms (Fig. 8 *B*, *Lower*). However, the correlation effects associated with these pulses were more short-lived, generally dying away within 40 ms. In contrast, responses from the LIFG to LMTG were relatively slower but long-lasting, characterized by delays >20 ms and sustained effects as long as 100 ms (Fig. 8 *B*, *Upper*). In addition, while the LIFG to LMTG effects were somewhat stronger after the noun RP, clear evidence of information flow from the LIFG to LMTG already can be seen at noun onset, suggesting that early processing of the DO noun may be subject to LIFG-generated cognitive control.

Although the regions in the directed connectivity analyses were selected based on their significant effects for particular model RDMs, this method is still largely data-driven. Therefore, 2 additional sets of control analyses were conducted to investigate whether our findings are specific to speech comprehension or simply driven by intrinsic interactions between brain regions. The results support the former possibility (*SI Appendix*, section 3).

## Discussion

In this study, we investigated the neural mechanisms underpinning semantic composition—the rapid combinatorial processes that support integration of the meanings of successive spoken words in an utterance, and the ways in which the meaning of one word affects the interpretation of an upcoming word during real-time incremental speech comprehension. The specific instance we focused on concerns how a DO noun is flexibly interpreted in the context of the preceding verb in a short sentence. Given our focus on semantic composition, we held the syntactic context constant, using the same simple sentential structure across all the stimulus materials. In what follows, we lay out the framework that emerges from this study, providing spatiotemporally well-specified insight into the qualitative and quantitative properties of the neural processes that underpin core aspects of incremental interpretation.

### Accessing and Integrating Verb Semantics.

Developing an account of how verb semantics interacts with the semantic properties of the following DO noun first requires an understanding of how the relevant semantic properties of the verb are themselves activated and made available as constraints on subsequent words. These processes were assessed here using 2 model RDMs based on topic modeling estimates of verb semantics: the verb topic RDM and verb topic entropy RDM. The model fit for these RDMs across the verb epoch, as summarized in Fig. 6 *A* and *B*, implicates a network of regions across the left temporal lobe from the LATL to the posterior temporal cortex and extending dorsally into the SMG and AG, with the strongest model fit seen in the LpMTG and SMG/AG. The verb topic RDM in particular engages the LpMTG throughout the verb epoch (Fig. 6*A*). The nature of the processing interactions between these regions is illuminated by the directed connectivity analyses during this epoch (Fig. 8*A*).

The verb topic RDM captures the representational content of verb semantic constraints. It shows a weak early model fit in the LMTG from verb onset, with stronger effects emerging around 100 ms later. Model fit spreads from the initial focus in the LpMTG to both the LATL and L SMG/AG around verb RP, as the verb is being recognized. The directed connectivity between the LpMTG and L SMG/AG—the 2 regions showing the strongest model fit to the verb topic RDM (Fig. 6*A*)—suggests that information flow originating from the LpMTG is continuously delivered to the L SMG/AG at very short delays (generally around 20 ms) throughout the verb epoch (Fig. 8 *A*, *Lower*). In contrast, information flow in the opposite direction, from the L SMG/AG to LpMTG, is much more intermittent and does not begin until verb RP, 300 ms after verb onset (Fig. 8 *A*, *Upper*). These patterns of connectivity suggest that information about verb semantic content is continuously generated in the LpMTG as the speech input accumulates (34, 45–47) and is continuously delivered to the L SMG/AG (among other regions) for further integration, consistent with the widespread view that the L SMG/AG plays an important role in semantic integration at both phrasal and sentential levels (10, 11, 48–50). The timing of information flow from the SMG/AG to LpMTG, occurring only as the verb is recognized, suggests that this reflects modulation of lexical analysis activities in the LpMTG, triggered by the integration of verb semantic properties into the current utterance representation.

The critical role of verb RP, where the semantics of the actual verb come to dominate the neural response to different models, is reflected in the timing of model fit for the verb topic entropy model (Fig. 4*B*). This model RDM reflects not the representational content of the verbs, but rather how constraining that representation is. A verb with preferences for fewer topics is more constraining and thus has lower entropy, resulting in less uncertainty about the likely properties of the following DO noun. This information is critical for processes of incremental combination, since it determines how strongly different semantic constraints can be placed on the upcoming word. These entropy values can only be computed once the topic distribution of the actual verb is known, and it is precisely around verb RP that the model fit for this RDM is first seen (Fig. 4*B*). Consistent with this account and the proposed role in semantic integration for the LATL and L SMG/AG (50), the topic entropy RDM shows a strong model fit in these 2 regions as well as in the LpMTG (Fig. 6*B*).

Finally, when considering the semantic constraints projected by the verb (in the context of its preceding subject noun) on the following DO noun, it is important to define the likely nature of these constraints. Given that a topic is a probabilistic distribution over the whole vocabulary of DO nouns rather than specific semantic features of a concept, the constraints represented by the verb topic vector typically take the form of general semantic categories such as “food” rather than specific entities such as “bread.” This suggests that a broad semantic representation that shares the topics preferred by the verb is generated after the verb has been recognized. This broad semantic set is then used to guide interpretation of the following DO noun (51). In fact, the topic vector may represent semantic structure in terms of category organization, with a topic representing, for example, concepts relating to a food, plant, or animal and so forth, which provides a plausible account for the involvement of the left posterior inferior temporal cortex for the verb topic effects, given its important role in processing categorical semantic information (47, 52, 53).

### Contextual Constraints in Semantic Combination.

Turning to the DO noun epoch and the access of noun semantics as the noun is heard, we addressed the controversial issue of how the meaning of an upcoming word is modulated by its context (25–30). The combination of topic modeling, EMEG source space, and ssRSA allowed us to ask (and answer) this basic question about flexible meaning by constructing model RDMs of DO noun semantics that were either context-independent or context-sensitive and then determining which of these models showed a significant fit to neural activity as the DO noun was heard, and when and where these effects occur. The verb-weighted noun topic RDM contained only topics preferred by both a verb and its DO noun, while the noun topic RDM represented the full context-independent semantics of the noun.

The results reveal a striking contrast in model fit over the noun epoch. The verb-weighted noun topic RDM shows significant effects in the LIFG and LMTG, beginning around the noun RP and continuing through to noun offset (Figs. 5*A* and 6*C*), but the context-independent noun topic RDM shows no significant model fit at any point throughout the noun epoch (Fig. 5*B*). This seems to provide direct evidence that the DO noun is flexibly interpreted in the context of the semantic preferences of its preceding verb. In first-pass processing of the speech input, the semantic properties of the word that are not prioritized in the prior context are either only very weakly activated, such that they are not detected by the methods used here, or else not activated at all.

The issue of how DO noun semantics is selectively activated was addressed by 2 other model RDMs designed to probe different aspects of the processes supporting semantic combination. These are the verb and noun interaction RDM (Fig. 5*C*), designed to identify the processing mechanisms involved, and the verb constraint error RDM (Fig. 5*B*), which taps into the variations in processing activity generated by the process of integration itself—the contact between noun semantic representations and the semantic preferences projected by the preceding verb. Both models, like the verb-weighted noun model, show a strong model fit in the LIFG, in all cases either around or after noun RP.

The LIFG is widely considered a key region for semantic retrieval (47), especially for the controlled selection of semantic knowledge (54, 55), and it plays a central role in semantic integration in the MUC (memory, unification, control) model (3–6). Different subdivisions of the LIFG are generally assigned different roles in semantic controlled processing, with BA45 likely more involved in selection and integration and BA 47 more engaged in semantic retrieval (41–43). Consistent with this, the verb-weighted noun topic RDM, which captures the contextualized semantic representation generated as the noun is heard, shows effects peaking in BA47 from noun RP to noun offset, with varying degrees of anterior and posterior L temporal engagement (Figs. 5*A* and 6*C*). In contrast, BA45 is more strongly engaged by the verb and noun interaction RDM, which generates model fit primarily in BA45 and extends to BA47 only later (Figs. 5*C* and 6*D*), while the verb constraint error RDM similarly shows model fit at noun RP for BA45 and extending into BA47 over the next 200 ms (Figs. 5*D* and 6*E*). This is consistent with a dominant role for BA45 in the control processes that select contextually relevant semantic properties (41). Note, however, that the peak effects of the verb constraint error RDM were found in the LpMTG (Fig. 6*D*), suggesting its strong involvement in representing the relevant semantic properties of the verb and its DO noun during the process of semantic combination.

The salient role of the LIFG in these noun epoch RDMs is reflected in the directed connectivity between the LIFG and LMTG (Fig. 8*B*). Despite the absence of model fits to the DO noun-relevant RDMs before DO noun RP, information flow between the LIFG and LMTG in both directions was found from noun onset. Similar to the pattern revealed during the verb epoch, information flow from the LMTG was rapidly updated with a delay of 20 ms, suggesting that retrieved lexical-semantic properties are immediately projected to the LIFG for further neural computations. This finding is consistent with the results of a recent MEG study that identified the middle temporal regions as an outflow hub sending widespread output to other language-relevant brain areas (56). In the opposite direction, information flowing to the left MTG is characterized by intermittent occurrence, longer delays, and relatively sustained effects as long as 100 ms. Importantly, however, directed connectivity effects from the LIFG to LMTG were already present at DO noun onset, implying that the semantic interpretation of the upcoming noun was already subject to probabilistic verb semantic constraints at noun onset. While neurocognitive models have highlighted the general role of the LIFG in combining individual words into larger units (3, 4), these directed connectivity results reveal the detailed spatiotemporal structuring of these processes.

### Conclusions.

In this study, we developed quantitative semantic models based on topic modeling and tested them against real-time brain activity recorded by source-localized EMEG using ssRSA, to reveal the spatiotemporal neural dynamics of how the prior semantic context drives the semantic interpretation of an upcoming noun. Further directed connectivity analysis revealed distinct temporal patterns of top-down and bottom-up information flow between critical language regions, which reveal the neural mechanisms underpinning an essential property of spoken language—our ability to combine sequences of words into meaningful expressions.

## Methods

### Participants.

Sixteen right-handed native British English speakers participated in this study (age 18 to 39 y, 10 females) and provided written consent. All participants had normal hearing, and none had any preexisting neurological conditions or mental health issues. This study was approved by the Cambridge Psychology Research Ethics Committee.

### Stimuli.

We constructed 60 sets of 6 spoken sentences of the form “SNP + verb + DO noun” (e.g., “The elderly man ate the apple”). Fourteen different human subjects (e.g., “man,” “neighbor”) were used to build SNPs modified by an adjective (e.g., “elderly man,” “next-door neighbor”), making them likely to be interpreted as the agent of the actions depicted by the verb. The frequent repetition of the same SNP (each repeated a mean of 25.7 ± 9.0 times) was intended to minimize their influence on the semantic interpretation of the verb. In contrast, to generate a wide range of variation in constraint between the verb and the DO noun across the stimulus set, 3 different verbs were selected for the 6 sentences in each set, and each verb was paired with 2 different concrete DO nouns, giving 360 sentences in total. Verbs were in the past tense, and there was a determiner (“the”) between the verb and its DO noun. All of the verbs used in this study had a strong preference for a DO complement phrase according to their subcategorization (SCF) distribution provided by VALEX (57) (average probability of DO SCF, 0.60 ± 0.17). Thus, we constructed sentences in which the combined meaning of the verb and DO noun was highly semantically transparent, in the sense that the semantic relationships between them are consistent with the syntactic structure (7), and where this syntactic structure (as simple active declarative sentences) was held constant across the stimulus set. For each DO noun, the 3 verbs varied in both the content and strength of their semantic constraints. Verb constraint strength was quantified by verb topic entropy (*SI Appendix*, Fig. S4 and described in more detail below). The lemma frequency, familiarity, and imageability were, respectively, 106.5 ± 240.4, 519.2 ± 72.5, and 456.3.5 ± 96.1 for the verbs and 55.1 ± 62.7, 559.9 ± 52.1, and 605.5 ± 31.1 for the DO nouns. Lemma frequency was obtained from CELEX (37), and familiarity and imageability were obtained from the MRC psycholinguistic database (58).

### Procedure.

Participants were required to listen attentively and to answer occasional questions that appeared on the screen in front of them with a response box to maintain alertness (treated as filler trials). These filler trials were excluded from the subsequent analyses. Instructions were visually presented on a monitor screen situated in front of the participant. Auditory stimuli were delivered binaurally through MEG-compatible ER3A insert earphones (Etymotic Research). There was a mean 26 ± 2 ms delay in sound delivery due to transmission of the auditory signal from the stimulus computer to participants’ ears. To ensure that participants were able to hear the stimuli through both earphones, a short hearing test was conducted before the main experiment.

The experimental stimuli (360 spoken sentences) were equally divided into 4 blocks with 90 experimental trials in each. To maintain participants’ attention, the experimental trials in each block were interspersed with 9 filler trials consisting of questions related to the preceding sentence. These questions were presented in written form on the monitor screen, and a “yes” or “no” response was required. Each filler trial was followed by an additional filler sentence to ensure that no residual task effects would be picked up in the next experimental trial. The orders of blocks and also of trials within blocks were pseudorandomized across participants. Each experimental trial began with a fixation cross presented at the center of the screen for 650 ms, which was followed by a variable gap (750 or 1,350 ms) before sentence onset. Participants were asked to avoid blinking while listening to sentences; there was 1,000 ms of silence at the end of each sentence, followed by a “blink” cue lasting for 1,400 ms, during which participants could blink. E-Prime Studio version 2 (Psychology Software Tools) was used to present the stimuli and record the participants’ responses.

### Gating Pretest.

We used a behavioral gating task (39, 40) to determine the RP of the DO noun in the sentential context. The RP is the point in the speech input at which the word can be uniquely differentiated from its phonological competitors and thus the point at which the word is recognized (36). Twenty-four native British English speakers (age 18 to 40 y) who did not participate in the main experiment were recruited for the gating test. The same sentences used in the main experiment were presented in 50‐ms segments from the onset of the DO noun. For example, participants heard *“*The elderly man ate the...”, “The elderly man ate the a...”, “The elderly man ate the app...” over headphones in a sound-attenuated room. They were required to provide a continuation word, with a confidence score scaled from 1 to 7 (where 1 = not confident at all and 7 = very confident). The same sentence was repeated with increasing increments of 50 ms until the participant provided the same response with a confidence score of 7 twice. Noun RP was defined as the gate where 80% of participants gave the correct response twice in a row.

### EMEG and MRI Acquisition.

Participants were seated in a magnetically shielded room (IMEDCO) with the head placed in the helmet of the MEG scanner. MEG data were collected using a Neuromag Vector View system (Elekta) with 102 magnetometers and 204 planar gradiometers at a 1-kHz sampling rate. Simultaneous electroencephalography (EEG) was recorded at a 1-kHz sampling rate from 70 Ag-AgCl electrodes within an elastic cap (ESAcYcAP). Vertical and horizontal eye movements were recorded by 2 electrooculography (EOG) electrodes attached below and lateral to the left eye, and cardiac signals were recorded by 2 electrocardiography (ECG) electrodes attached separately to the right shoulder blade and left torso. Five head position indicator (HPI) coils were used to monitor head motion. A 3D digitizer was used to record the position of EEG electrodes, HPI coils and ∼100 to 150 head points on participants’ scalp relative to the 3 anatomic fiducials (i.e., nasion and bilateral preauricular points). To source localize EMEG data, T1-weighted MPRAGE structural magnetic resonance imaging (MRI) with 1-mm isotropic resolution was acquired using a Siemens Prisma 3-T scanner. All EMEG and MRI data were collected at the MRC Cognition and Brain Sciences Unit, University of Cambridge.

### EMEG Preprocessing and Source Localization.

Maxfilter (Elekta) was applied to raw MEG data for bad channel removal and head motion compensation. Signals outside the brain were removed using the temporal extension of signal-space separation (59). EMEG data were then down-sampled to 500 Hz. Independent component analysis (ICA) was conducted using EEGLAB, and components related to blink, eye movement, and physiological noises were removed according to the correlation with EOG and ECG signals and further visual inspection. The following preprocessing steps were conducted using SPM12. A low-pass fifth-order bidirectional Butterworth filter at 40 Hz was applied to ICA-deartifacted EMEG data. Two epochs were extracted from continuous data with auditory delivery delay corrected; one was aligned to verb onset and extended to 600 ms afterward, and the other was aligned to noun onset and extended to 640 ms afterward. Epoch length was determined by the summation of the mean ± 1 SD of the duration of the verb or DO noun speech input (verb, 487 ± 116 ms; DO noun, 523 ± 114 ms). Baseline correction was performed by subtracting the time-averaged signal of a silent period (i.e., −200 ms to 0 ms relative to sentence onset) from the epoched data. Finally, automatic artifact rejection was conducted to exclude trials with signals that exceeded amplitude thresholds (60 ft/mm for gradiometers, 3,000 ft for magnetometers, and 200 μV for EEG electrodes). The mean ratio of rejected trials was 4.5% for the verb epoch and 5.2% for the noun epoch.

EMEG data source localization was performed using SPM12. Source space was modeled by a cortical mesh consisting of 8,196 vertices. The sensor positions were coregistered to individual T1-weighted structural images by aligning fiducials and the digitized head shape to the outer scalp mesh. The MEG forward model was constructed using the single-shell model (60), and the EEG forward model was built using the boundary element model (61). Inversion of EMEG data was performed for verb epoch and noun epoch separately using the least squares minimum norm method (62) and an empirical Bayesian MEG and EEG data fusion scheme (63) implemented in SPM12. In general, MEG is insensitive to radially oriented sources, which are prominent in EEG, while EEG suffers from relatively lower spatial resolution in source localization due to distortion caused by heterogeneous electrical conductivity through the skull and scalp. The combination of EEG and MEG gives more accurate reconstructions by integrating the complementary information provided by the 2 modalities (63–66).

### Topic Modeling.

Topic modeling was adopted to quantify verb and DO noun semantics using the LDA algorithm (31). LDA is a generative probabilistic model originally proposed to discover the latent semantic topics within massive collections of documents (18). Topics are represented by multinomial distributions over the whole vocabulary consisting of words from all documents in large-scale corpora. The generative process of topic modeling assumes that each document is created by first being assigned with a distribution over topics, and then each word in this document is chosen from a topic selected according to this document’s distribution over topics. The training of LDA aims to reveal the hidden topics and each document’s distribution over topics.

Given the distributional hypothesis of semantics—that is, words that are used and occur in the same contexts tend to have similar meanings (67, 68)—LDA was used to quantify a verb’s semantic constraints based on its frequency of co-occurrence with DO nouns. Specifically, we used the Local Mutual Information (LMI) from the Distributional Memory tensor (32), which is calculated based on the raw co-occurrence frequency count between a verb and its DO noun and has considerable computational advantages, including avoiding bias toward overestimating the significance of low-frequency items. Based on the co-occurrence frequency (i.e., LMI value) between a verb and its DO nouns, we can construct a verb document that includes all the DO nouns of this particular verb. In such a verb document, each DO noun is repeated *N* times, where *N* is the co-occurrence frequency between the verb and this DO noun. Thus, a verb document depicts the semantic constraints of this verb through the DO nouns with which it co-occurs in large-scale corpora. The training of LDA was restricted to the relationship between a verb and its DO nouns, with the intention of focusing on semantic modeling by keeping the syntactic structure constant (i.e., verb and DO noun). Note that although the verb document is not a realistic document, the verb and DO noun co-occurrence embedded in it are indeed extracted from real corpora containing 2.83 billion tokens (32). The training data set consisted of 4,217 verb documents (all transitive verbs with a nonzero DO SCF probability according to VALEX) with a vocabulary of 20,373 DO nouns (92.5 million tokens) from the corpora. The topics inferred from these verb documents constitute a semantic space in which each verb’s semantics can be characterized by a verb topic vector, that is, the unique distribution over topics given a verb, P(topic|verb). On the other hand, the multinomial distribution of topics provides the probability of each DO noun given a certain topic, P(DO noun|topic). By applying the Bayes theorem, we can also obtain the distribution over topics given a DO noun:

P(topic|DO noun)=P(DO noun|topic)×P(topic)/P(DO noun).

Thus, noun semantics can be represented by a noun topic vector. By doing this, verb and noun semantics were represented using the same set of topics.

Topic modeling was conducted using an open-source implementation of Bayesian variational method for LDA (https://github.com/blei-lab/lda-c). The optimal number of topics was determined by evaluating the results for topic models with different topic numbers (*SI Appendix*, section 4). As mentioned above, each topic is a distribution over the whole vocabulary from the corpora; however, the degree of semantic dispersion can vary across topics, potentially undermining the estimation of topic entropy (see definition in *Cognitive Models*). For example, the entropy of a verb with less specific semantic constraints (e.g., “want,” “like”) could be underestimated if the uncertainty of its constraints were reflected by the preference for only a few less informative topics (i.e., a more concentrated pattern over topics), which leads to a low entropy value. We quantified the informativeness of each topic and applied it to the loading of this topic in both verb and noun topic vectors to alleviate the semantic dispersion across topics (*SI Appendix*, section 5).

### Cognitive Models.

A series of computational cognitive models were constructed using verb and noun topic vectors obtained from LDA. Verb topic vectors provide information about both the content of constraints (i.e., which topics are preferred) and the strength of constraints (i.e., whether they show a focused or distributed pattern over topics). The strength of semantic constraints can be isolated by calculating the entropy embedded in the verb topic vector,

$$H\left(v\right)=-\sum_{i}P_{i}\cdot log\left(P_{i}\right),$$

where $P_{i}$ is the probability (i.e., normalized loading) of the i th topic for verb v.

The verb topic RDM was constructed by calculating the cosine distance between verb topic vectors, while the verb topic entropy RDM was a difference matrix constructed by calculating the absolute difference between the entropy values of verb topic vectors. The noun topic RDM, which captures the semantics of DO nouns, was constructed by calculating the cosine distance between noun topic vectors. To model the verb-constrained DO noun semantic representation, we built the verb-weighted noun topic RDM through element-by-element multiplication between verb topic vector and noun topic vector. Thus, within the noun topic vector, only topics preferred by both the verb and the DO noun are preserved, while those irrelevant to the verb are suppressed. The cosine distance between verb-weighted noun topic vectors was used to construct the verb-weighted noun topic RDM, which captured the semantic representation of a DO noun in the context of the preceding verb.

In a further analysis, we also partialled out both verb and noun topic RDMs from the verb-weighted noun topic RDM on the hypothesis that any remaining effects would be due to the interaction between the verb and DO noun semantics. Finally, we quantified the ease of fitting the noun into the semantic constraints of the preceding verb by calculating verb constraint error, defined as the cosine distance between the verb topic vector and noun topic vector. The smaller the verb constraint error, the easier it is to fit the noun into the verb semantic constraints. The verb constraint error RDM was a difference matrix constructed by calculating the absolute difference between verb constraint error values of different verb and DO noun combinations. All the model RDMs described above had the same matrix size (360 × 360), and each off-diagonal element indicates the dissimilarity between 2 of the 360 spoken sentences to which the participants were exposed.

### ssRSA.

The ssRSA method combines both temporal and spatial multivariate patterns to reveal the neural substrates underlying cognitive processes by correlating the dissimilarity generated by cognitive models with the dissimilarity generated by the corresponding brain activity (19, 20). We used a spatiotemporal searchlight with a 10-mm spatial radius and 30-ms temporal radius (i.e., a 60‐ms sliding time window), which was mapped across the source space of EMEG. The ssRSA analysis was restricted to a bilateral language mask that covered regions that have been consistently reported in studies on language processing, including the bilateral temporal cortex, inferior frontal gyrus, supramarginal gyrus, and angular gyrus (33–35). To construct data RDMs for each searchlight, we composed data vectors by extracting source-localized EMEG data corresponding to each of the 360 spoken sentences and calculated the pairwise Pearson correlation distance (i.e., 1 − Pearson *r*) among them, which resulted in a 360 × 360 data RDM. Multivariate normalization was applied to the data RDMs to improve the reliability of distance measures and reduce the task-irrelevant heteroscedastic structure across trials and vertices (69). The data RDM of a searchlight centered at each vertex and time point was compared against the cognitive model RDMs using Spearman’s rank correlation, which resulted in a time course of model fit for each vertex. In the verb epoch, we tested verb topic RDM and verb topic entropy RDM. In the noun epoch, we tested verb-weighted noun topic RDM, noun topic RDM, verb–noun interaction RDM (partialling out both verb and noun topic RDMs from verb-weighted noun topic RDMs), and verb constraint error RDM. For each time point, a 1-tailed 1-sample *t* test was conducted at each vertex with the fits of all participants for 1 model RDM to test whether the mean model fit is larger than 0. Cluster permutation tests were performed for multiple comparison correction with 5,000 nonparametric permutations (38), vertex-wise *P* < 0.01 and cluster-wise *P* < 0.05.

### Information Flow between Brain Regions.

To reveal how information is transferred between brain regions, we calculated directed connectivity based on the data RDMs of 2 regions that showed significant model fits for a specific model RDM (21, 22). The logic is that if region A has causal effects on region B, then the activity of A at a previous time point can be used to explain the current activity in B better than simply using the previous activity of B alone. We define the data RDM of region X at time point *t* as D(X, *t*); the directed connectivity from A to B is quantified as the partial correlation coefficient between D(A, *t* − *dt*) and D(B, *t*) partialling out D(B, *t − dt*), where *dt* is the time interval between the current time point and the previous time point used to calculate directed connectivity. To avoid bias due to the choice of dt, we calculated directed connectivity with a series of dt values ranging from 2 ms to 120 ms, which precisely described the onset and duration of the directed connectivity between 2 brain regions. Note that data RDMs were recalculated by only using data at each time point instead of that within a sliding time window, to avoid contamination from neighboring time points. Regions of interest were determined by selecting the 100 most significant vertices, as quantified by the summation of *t* values (for a particular model RDM) at each significant time point within an epoch, restricted to the anatomic areas defined by the automated anatomic labeling template (70).

### Data Availability.

Dataset relevant to this study is available at ref 71.

## Supplementary Material

Supplementary File
