# Supplementary material for: Neural dynamics of semantic composition
Source: Proc Natl Acad Sci U S A. 2019 Sep 30;116(42):21318–27. doi: 10.1073/pnas.1903402116 (PMC6800340; doi:10.1073/pnas.1903402116)
Supplement: Supplementary File [file pnas.1903402116.sapp.pdf]

PNAS
www.pnas.org

Supplementary Information for

Neural dynamics of semantic composition

Bingjiang Lyu, Hun S. Choi, William D. Marslen-Wilson, Alex Clarke, Billi Randall and Lorraine K. Tyler

Lorraine K. Tyler
Email: lktyler@csl.psychol.cam.ac.uk

Table of Contents

1. Overlap between the semantic constraints of subject noun and verb

2. Distribution of verb topic entropy

3. Additional connectivity control analyses

4. Number of topics

5. Semantic dispersion across topics

6. Illustrative topic modelling results

www.pnas.org/cgi/doi/10.1073/pnas.1903402116

1. Overlap between the semantic constraints of subject noun and verb

We summarise here the further analyses conducted to investigate the possible sources of the model fit seen for the verb topic RDM from verb onset. We focus in particular on the shared semantic properties of the subject noun and the following verb.

Psycholinguistic studies have shown robust priming effects of agents (or subject nouns) on the verbs with which they typically occur (1, 2). The authors explain this in terms of event knowledge, that is, information about real-world events triggered by the agent and verb in a sentence is dynamically combined by comprehenders. Consistent with this account, the congruency across words also facilitates online sentence interpretation (3). In the current study, one aspect of the congruency between the subject noun and the verb is the semantic constraints they have in common over DO nouns. In other words, a subject noun and a verb can be linked to each other in an event structure because they tend to occur with similar DO nouns. The use of computational modelling methods (e.g., LDA) enables us to quantity this relationship through statistics-based measures from large-scale corpus (e.g., co-occurrence frequency).

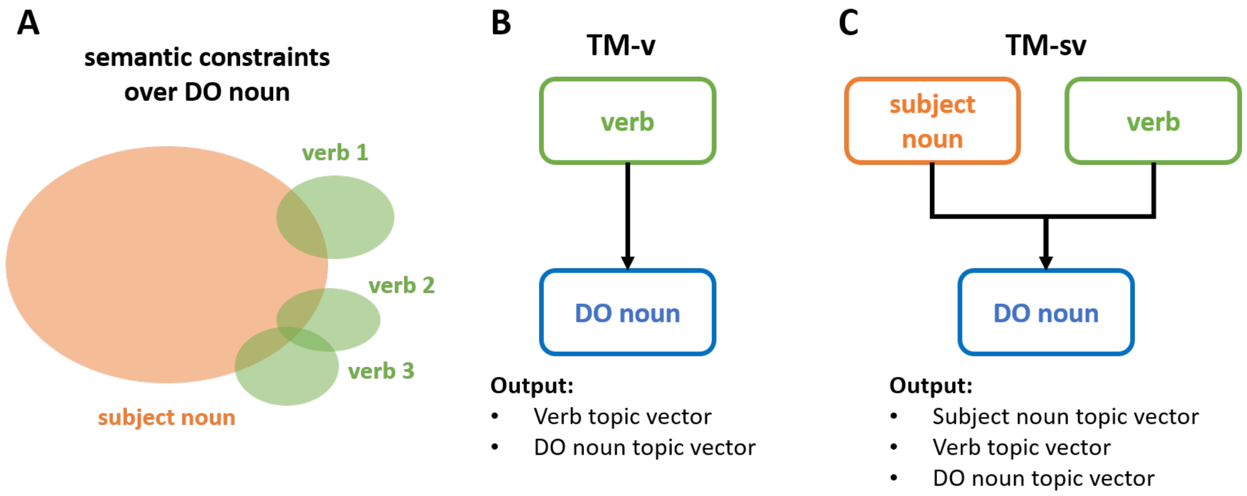

Figure S1 *Illustrations of (A) the relationship between the semantic constraints over the DO noun from the subject noun and the verb, (B) topic modelling of verb's semantic constraints and (C) topic modelling of both subject's and verb's semantic constraints over the DO noun (TM = Topic Model).*

Given that a subject noun and a DO noun can co-occur with different intervening verbs, a subject noun is likely to provide relatively broader semantic constraints over DO nouns in contrast to a verb (see Figure S1A for illustration). Thus, we hypothesize that the early model fit of verb topic RDM is due to semantic constraints that are congruent across the subject noun and the verb. These should be activated once the subject noun is recognised (4, 5) as part of the subject noun's broad semantic constraints.

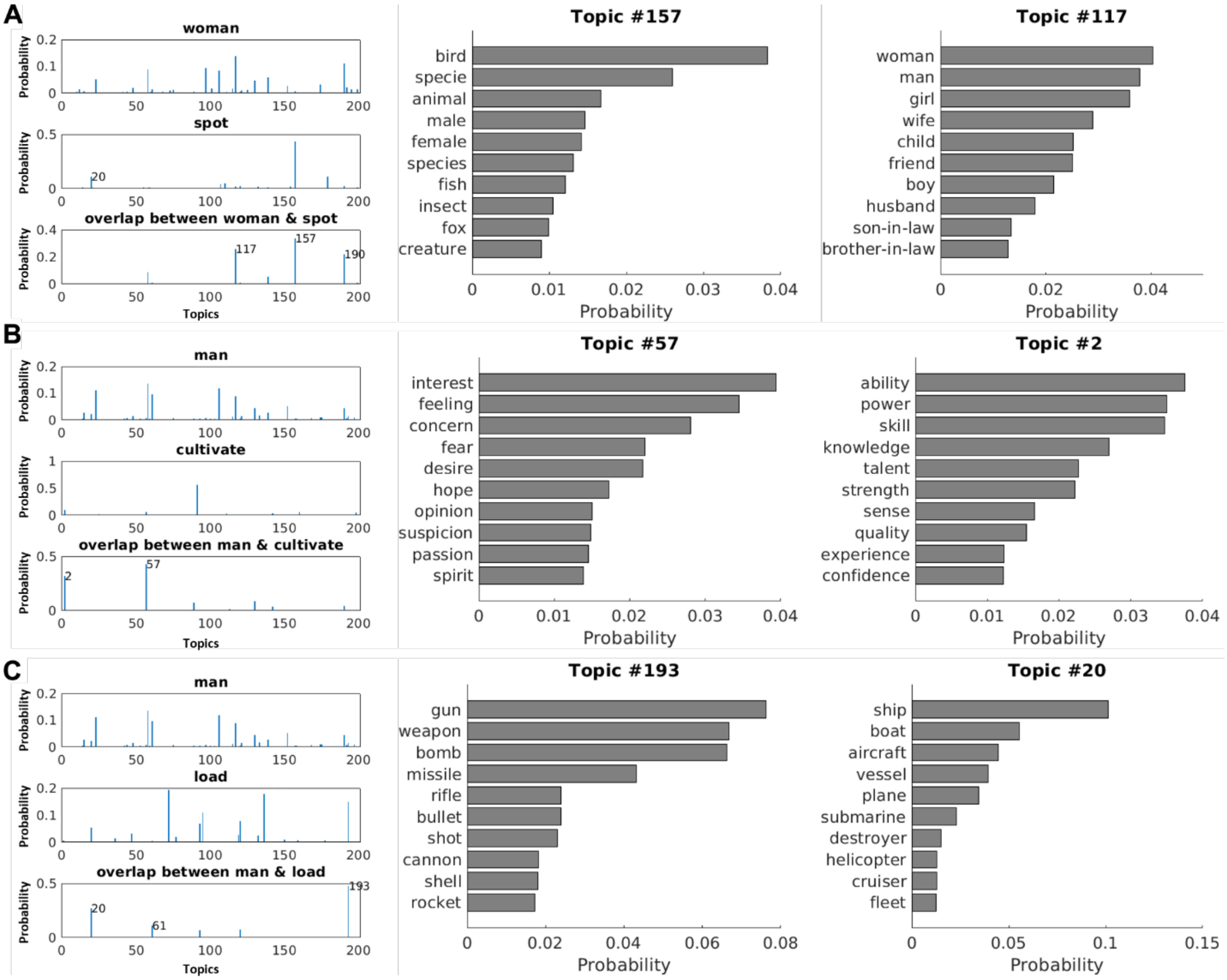

Figure S2 *Illustrative results from the topic model that quantifies both subject nouns' and verbs' semantic constraints over DO nouns (TM-sv). Results of three pairs of subject noun & verb are shown: (A) 'woman' & 'spot', (B) 'man' & 'cultivate', (C) 'man' & 'load'. The left column in each subplot shows, from top to bottom, the subject noun topic vector, the verb topic vector, and the subject noun & verb overlap topic vector obtained through element-wise multiplication (re-normalized for illustration purpose). The right two columns show the two most preferred topics in the subject noun & verb overlap topic vector, with each topic being represented by its ten most probable words.*

To test this hypothesis, we trained a topic model including both the subject noun and the verb to quantify their semantic constraints over the DO noun simultaneously (15,629 subject nouns and 4,217 verbs, Figure S1C). This is different from the topic model built on the verb-DO noun co-occurrence only, as reported in the main text (Figure S1B). For simplicity, these two topic models are referred to below as TM-sv and TM-v respectively. In TM-sv, the subject noun topic vector exhibits significantly higher topic entropy than the verb topic vector (subject noun: 2.65±0.55, verb:

2.21±0.92, two-tailed two-sample t-test, $t = 7.86$, $p = 1.5e-14$), suggesting that the subject nouns used in this study indeed provide less specific semantic constraints compared with the verbs. See also the relatively more distributed pattern in subject noun topic vectors and the sparser pattern in verb topic vectors in Figure S2. Note that we are not able to model the entire subject phrase (i.e., modifier and the subject noun) due to the limitations of current topic modelling methods.

We further constructed subject noun & verb overlap topic vectors through element-wise multiplication between the subject noun and verb topic vectors in TM-sv, capturing their shared semantic constraints. Although all subject nouns used in this study are human agents and the verbs are actions likely performed by humans, the shared semantic constraints between the subject noun and the verb include but are not limited to animacy, as shown in Figure S2. Moreover, the topic distribution of the shared semantic constraints varies across subject noun and verb pairs, providing the variance needed to construct a valid model RDM.

We extended the original verb epoch reported in the main text (aligned to verb onset) 50ms back into the subject noun, then tested several RDMs based on the results from TM-sv. Model fit for the 'pure' subject noun topic RDM is already present in bilateral temporal regions 50ms before verb onset, but these effects dissipate within 100ms after verb onset (Figure S3A). The subject noun & verb overlap topic RDM (TM-sv) shows similar left hemisphere effects before and immediately after verb onset, but model fit now continues until 224ms after verb onset with peak effects found in L MTG at 198ms, terminating well before verb recognition point (RP), estimated at 339 ms (Figure S3B). The results suggest that subject noun semantics remained activated as the verb begins to be heard but die off as the semantics of the new cohort, triggered following verb onset, begin to be activated. Consistent with this account, the more selective subject noun & verb overlap topic RDM, which contains only those subject noun topics that are also likely to be preferred by the verb, exhibits a longer-lasting model fit but also dies off well before verb RP. This drop off in model fit presumably reflects the growing activation of verb-specific semantics as the system begins to converge on the exact word being heard, so that the correlational structure in the data RDM of neural activity will change in ways that no longer match a correlational structure in a model RDM based only on subject noun/verb overlap. For example, the verb 'ate' in the context 'the elderly man …' will begin to evoke semantic properties related to 'eating' and its probable DO nouns that are not encoded in the semantics of the subject noun. Model fit for the two verb topic RDMs (based on TM-v and TM-sv separately) is already present 50ms before verb onset and remains evident throughout the verb (Figure S3 C&D) with similar spatiotemporal distributions, as expected given the high correlation between the two RDMs (Spearman's $r = 0.81$).

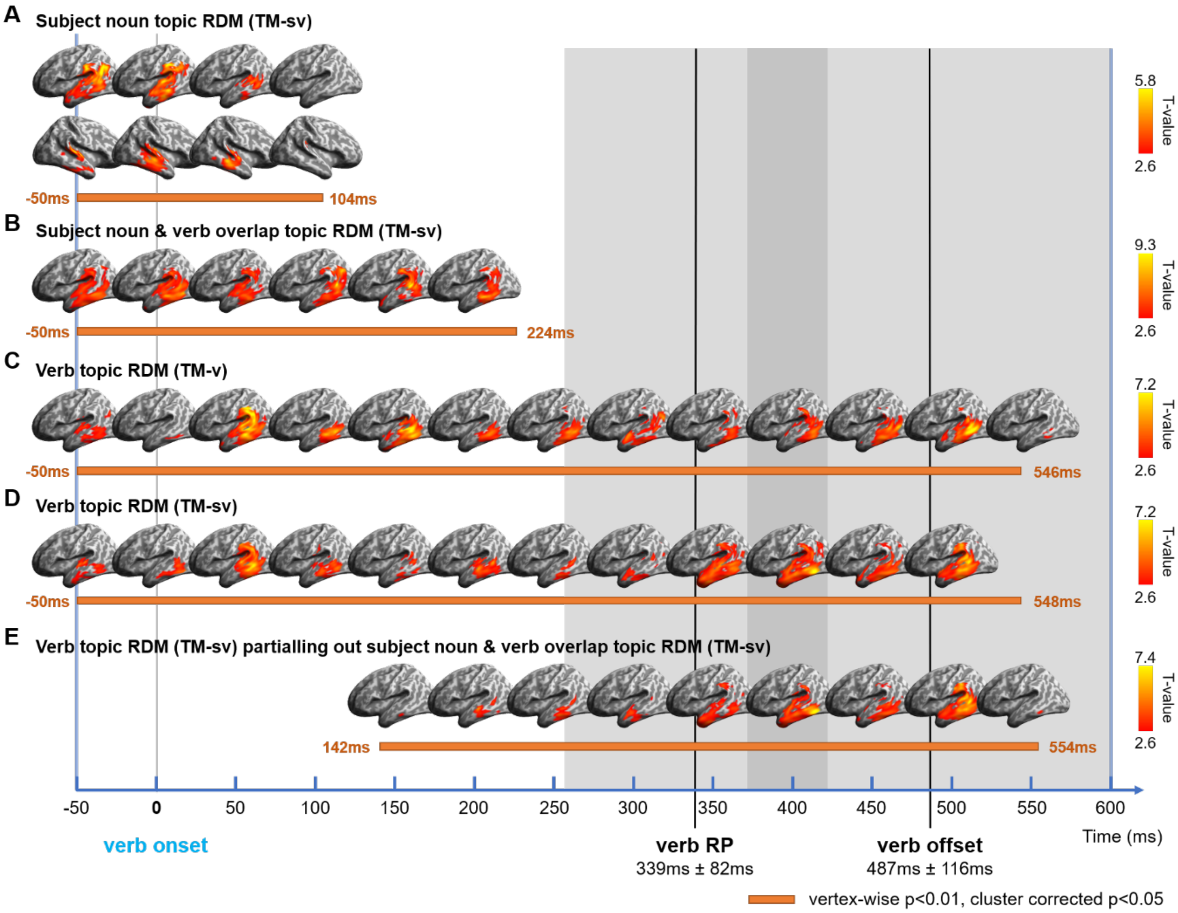

***Figure S3** ssRSA results for different model RDMs across the verb epoch. The subject noun topic RDM (A) is based on the TM-sv topic model (see text); the subject noun & verb overlap topic RDM (B) captures the shared semantic constraints between the subject noun and the verb; the two verb topic RDMs (C&D) are based on the TM-v and the TM-sv models respectively - the results for the TM-v topic model are provided for comparison; the final RDM (E) shows the effects on verb topic RDM model fit (for D) of partialling out the semantic constraints shared by the subject noun and the verb (as in B). TM-sv indicates the topic model trained by both the subject noun's and the verb's co-occurrence with the DO noun, TM-v indicates the topic model trained by only the verb's co-occurrence with the DO noun.*

To evaluate more directly these claims about the timing and the relationship of subject noun and verb-specific semantic constraints as the verb is heard and recognised, we conducted a further analysis in which we partialled out the subject-noun & verb topic overlap RDM (TM-sv) from the verb topic RDM (TM-sv). As shown in Figure S3E, significant effects of the verb topic RDM were not found until nearly 150ms after verb onset, while the later model fit, especially after verb RP, remains largely the same, suggesting that the early model fit seen for the verb topic RDM was primarily driven by shared subject noun and verb constraints.

Note that, apart from the 4,217 verbs involved in TM-v, TM-sv also includes 15,629 subject nouns, therefore the final set of topics forTM-sv could be considerably biased to account for the subject nouns' broad semantic constraints. This is why we kept the original results based on TM-v in the main text since the primary focus there was on the meaning composition between the verb and the DO noun. TM-sv is only trained and used to examine the subject noun's potential effects during the early processing of the following verb. Importantly, the effects of model RDMs related to the subject noun ended well before verb RP (Figure S3 A&B), while the effects of the verb topic RDM after verb RP remain almost the same after partialling out the shared semantic constraints (compare Figure S3 D&E), suggesting that the meaning composition between the verb and the DO noun is not strongly affected by the subject noun.

In the context of the above discussion and these additional analyses, we argue that early model fit to the verb topic RDM should be regarded as **topic-based** rather than word-based, in the sense that congruent or consistent topics remain activated throughout consecutive words, such as a subject noun and its following verb. It is these shared topics that support the continuing model fit with subject noun semantics as the verb begins to be heard, and that, in the same way, support the model fit seen for verb topic semantics over the same period and beyond. Clearly verb-specific information does rapidly become dominant and the topic distribution activated by the subject noun is restructured and replaced as the verb input accumulates, as we see reflected in the first 200 ms or so after verb input begins. We note, however, that this account remains speculative without further research specifically designed to test these claims for partial topic-based model fit with word-level RDMs.

2. Distribution of verb topic entropy

As demonstrated in Figure S4, the verbs used in this study constituted a continuous and broad distribution in terms of the strength of their semantic constraints as measured by the verb topic entropy.

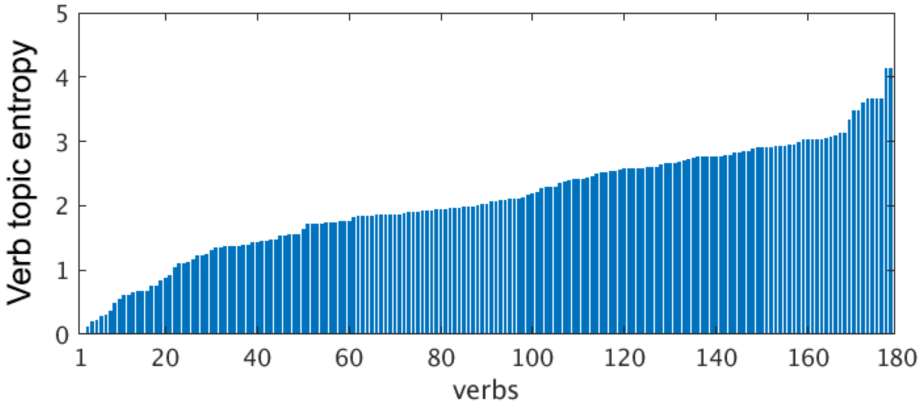

Figure S4 *Distribution of the strength of verb semantic constraints as measured by verb topic entropy.*

3. Additional connectivity control analyses

Although the ROIs selected in our directed connectivity analyses are determined by their significant model fit for a certain model RDM, this is still a largely data-driven method since the connectivity strength is quantified as the partial correlation coefficient between data RDMs. Therefore, we conducted two control analyses to investigate whether the connectivity results reported in the main text and above could be due to intrinsic brain-wide interactions and not specific to speech comprehension.

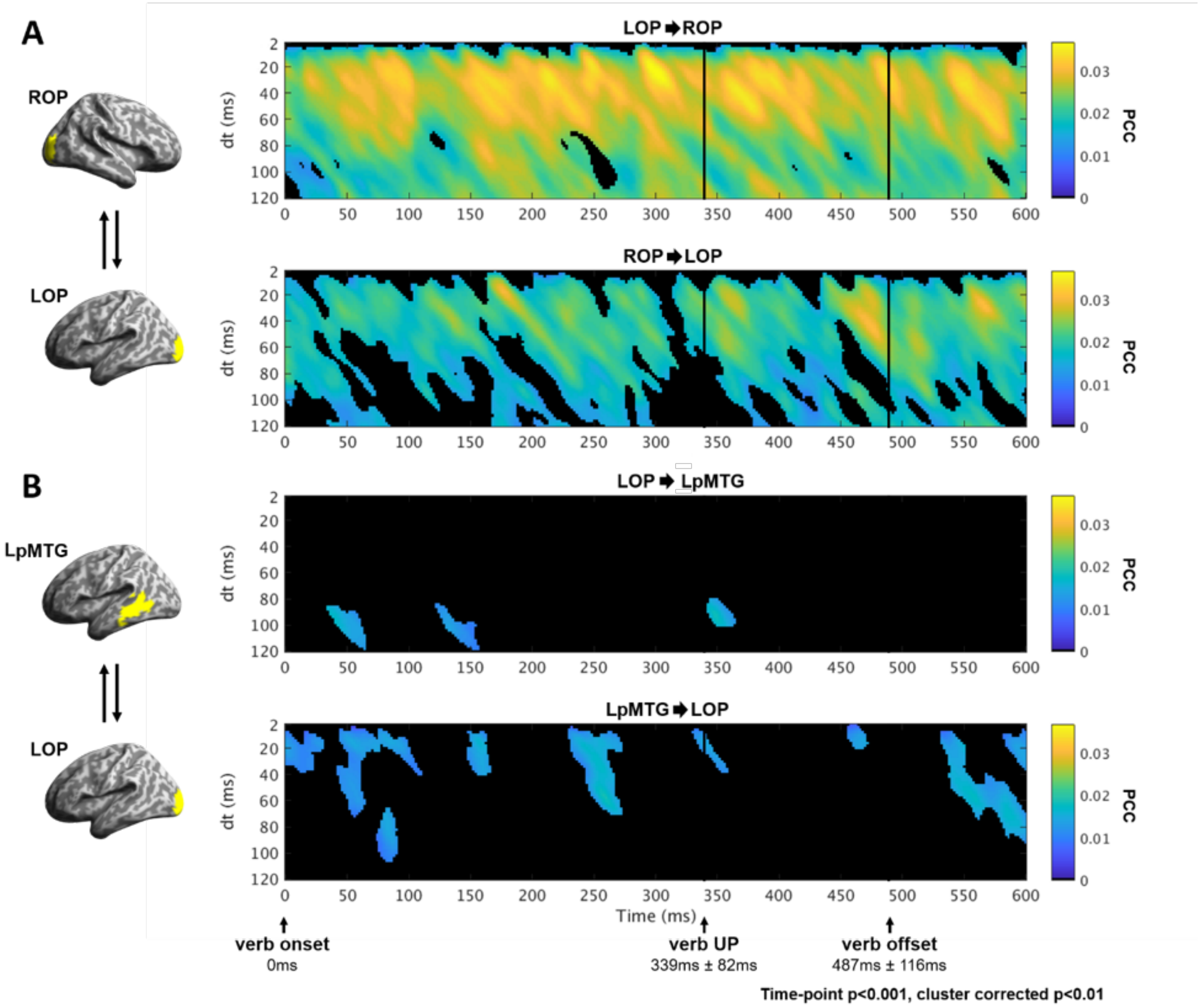

Figure S5 *Results of directed connectivity analysis between (A) the left occipital pole (LOP) and right occipital pole (ROP) during the verb epoch, and (B) the LpMTG and LOP during the verb epoch.*

Figure S5 gives the results of a test of the functional specificity of the directed connectivity results seen for the relationship between LpMTG and two language-related areas (LIFG and LSMG/AG), as plotted in Figure 8 in the main text. To do this we compared potential links between

LpMTG and the left occipital pole (LOP), assumed to have no direct functional relation, and between LOP and ROP, assumed to be strongly functionally connected to support visual processes. Consistent with these assumptions, LOP and ROP shows strong and continuous mutual connections, while no consistent connectivity is found between LOP and LpMTG. In other words, as one would expect, the patterning of data RDMs in LOP bear no significant statistical relation to future neural patterns in LpMTG.

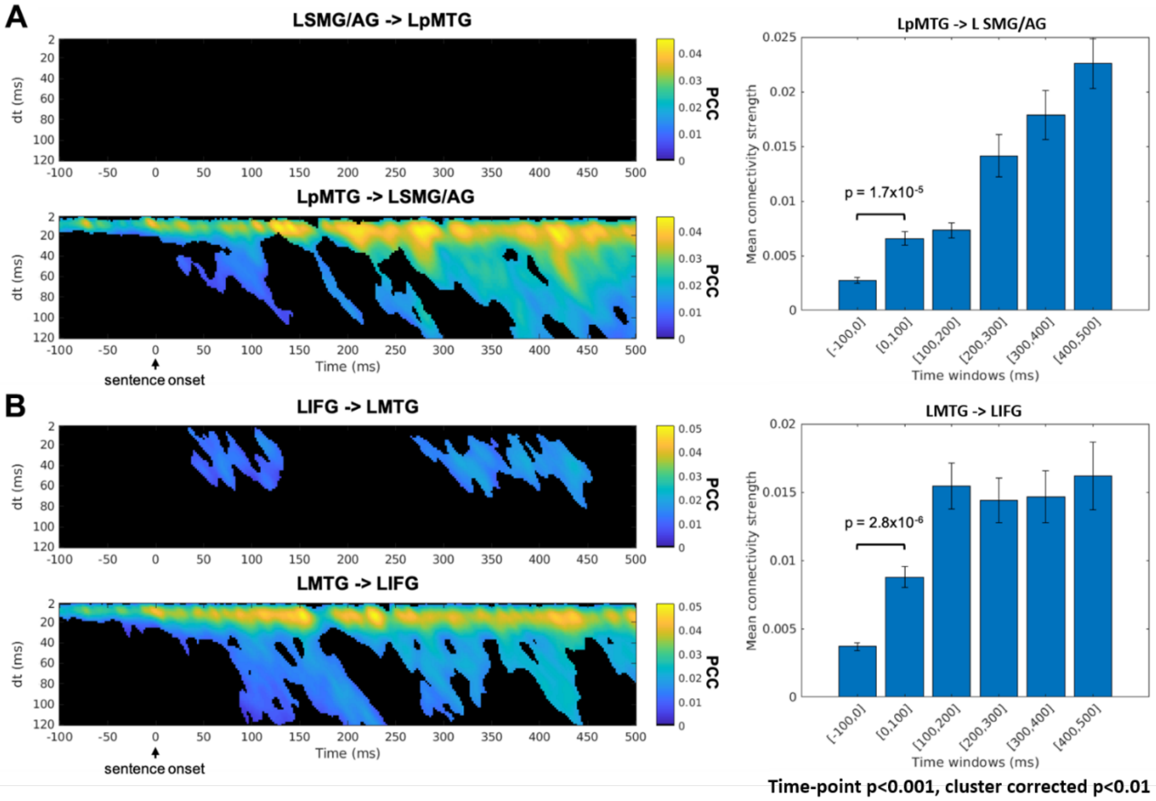

Figure S6 *Results of directed connectivity analysis between (A) L SMG/AG and LpMTG, and (B) LIFG and LMTG during an epoch aligned to sentence onset. Bar plots on the right side show the mean connectivity strength within successive 100ms time-bins from 100ms before sentence onset to 500ms after sentence onset.*

In the second control analysis (Figure S6) we tested the connectivity between relevant brain regions (as reported in Figure 8 of the main text) during an epoch aligned to sentence onset extending backwards 100ms. As shown in Figure S6, no effects are found before sentence onset, when there is no auditory input, both for the connectivity from L SMG/AG to LpMTG and from LIFG to LMTG. Although there are some early weak effects for the connectivity originating from the left MTG, consistent strong connectivity (i.e., the higher correlation plots in yellow) only emerges between 100-150ms after sentence onset when relevant speech input has started to accumulate.

To quantify this, we divided the epoch into six 100ms time-bins from 100ms before sentence onset to 500ms after onset, and averaged connectivity strength within each bin. It is clear from the bar graphs in Figure S6 that connectivity from LpMTG to LSMG/AG and LIFG is very weak in the pre-onset time-bin, starts to increase significantly in the first time-bin after sentence onset, but only reaches full strength after 100ms has been heard where the LIFG link is concerned, and 200-300ms for the SMG/AG connection. Note that the interval between trials is essentially a short silent period instead of a genuine resting state, so that we may well be picking up some residual effects from the last trial before the onset of the next trial.

4. Number of topics

The number of topics is a critical parameter that is set before model training. If the number of topics is too small, the topics obtained could cover a mixture of different categories, which means that these topics are less informative and more difficult to interpret. In contrast, if there are too many topics, a category might be decomposed into multiple topics, which results in redundant topics with similar meaning. Here we adopted topic coherence (6, 7) to choose an optimal number of topics. Topic coherence is often used for the overall evaluation of topic modelling results, moreover, it has been found to be more correlated to human judgement of interpretability than other measures (e.g., perplexity, the likelihood of unseen documents calculated using a trained model) (6). The rationale is that a good model will generate coherent topics that are informative and interpretable. For example, if the top 5 words of a topic are "apple, banana, orange, peach, mango", then this topic can be easily interpreted with a simple label "fruit".

In this study, we trained a series of topic models with different topic numbers (i.e., from 50 to 400 with increments of 50) and determined the optimal number of topics according to topic coherence. We used point-wise mutual information (PMI) as a metric of topic coherence (7). We calculated PMI using an independent external corpus (i.e., 5.2 million Wikipedia documents). The advantage of using an independent external corpus to evaluate topics is that it avoids reinforcing the noise and rare word relationship in the training corpus. For each topic t, PMI is defined as follow.

$$PMI(t) = \frac{2}{M(M-1)} \sum_{m=2}^{M} \sum_{l=1}^{m-1} log \frac{p\left(w_m^{(t)}, w_l^{(t)}\right)}{p(w_m^{(t)})p(w_l^{(t)})}$$

where $W^{(t)} = \left(w_1^{(t)}, w_2^{(t)}, \cdots w_M^{(t)}\right)$ is the top M words in topic t (here we used the top 10 words, i.e., M = 10), $p\left(w_m^{(t)}, w_l^{(t)}\right)$ is the co-occurrence probability of $w_m^{(t)}$ and $w_l^{(t)}$ in one document, $p(w_l^{(t)})$ is the occurrence probability of $w_l^{(t)}$ in one document.

As shown in Figure S7, both the mean and median of PMI scores peak at 200 topics, suggesting 200 as an optimal choice for the number of topics.

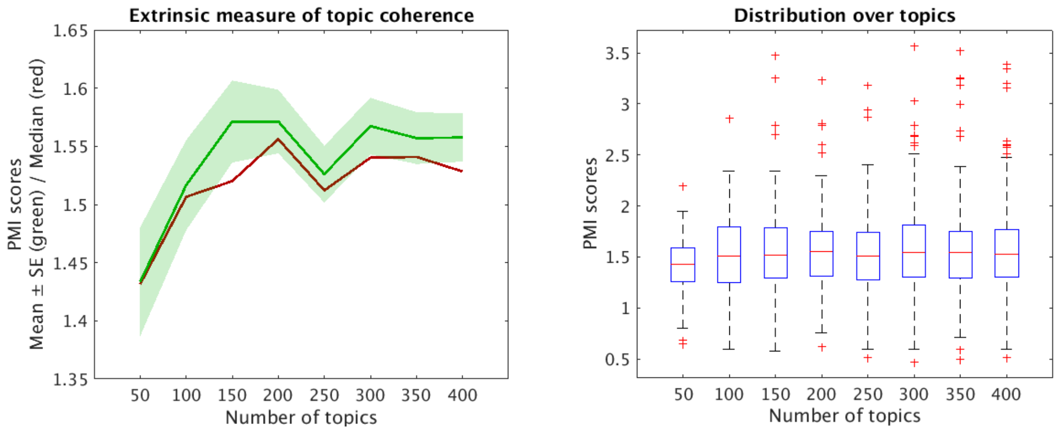

Figure S7 *Evaluation of topic models with different numbers of topics by topic coherence calculated from an independent external corpus (5.2 million Wikipedia documents). Left panel: green solid line and shadow indicate mean and one standard error (SE) across topics; red solid line indicates median value; Right panel: boxplots showing the distribution of PMI scores over topics. PMI, pointwise mutual information.*

5. Semantic dispersion across topics

The topics obtained from topic modelling differ from each other with respect to the extent of semantic dispersion, which could potentially undermine the estimation of verb topic entropy. Ideally, we would expect verbs with less specific semantic constraints to show higher verb topic entropy (i.e., a more distributed pattern over topics), and vice versa. However, the underlying assumption is that all the topics are equivalent with respect to the extent of semantic dispersion. Here informativeness is used to measure the semantic dispersion in each topic. The informativeness of topic t is defined as follow.

$$I(t) = \frac{2}{N_t(N_t - 1)} \sum_{i=1}^{N_t} \sum_{j=1}^{N_t} p(w_i|t) \cdot p(w_j|t) \cdot cos\left(\boldsymbol{n}_{w_i}, \boldsymbol{n}_{w_j}\right),\ (i < j)$$

where $p(w_i|t)$ is the probability of word w_i (from the training dataset vocabulary consisting of DO nouns) given topic t, $\boldsymbol{n}_{w_i}$ is the corresponding topic vector of w_i. In consideration of computational efficiency, we only included the N_t words with probability larger than 0.001 given topic t.

Thus high informativeness indicates the prominent words in this topic (i.e., words assigned with probability over 0.001) exhibit high semantic similarity, which corresponds to low semantic dispersion. Without semantic dispersion correction, the entropy of a verb with less specific constraints (e.g., *want*, *see*) could be underestimated if it only prefers a few less informative topics, which would result in a sparse distribution over topics and low verb topic entropy. We applied semantic dispersion correction by multiplying the informativeness of each topic with the original topic loading in verb/noun topic vectors (i.e., P(topic|verb), P(topic|DO noun)). As shown in Table S1, semantic dispersion correction mainly affects the verb topic entropy RDM, with very little effect on the other model RDMs.

Table S1 Spearman's r between model RDMs with or without semantic dispersion correction

Model RDM	Spearman's r between model RDMs with or without semantic dispersion correction
verb topic RDM	0.9633
verb topic entropy RDM	0.8337
noun topic RDM	0.9626
verb-weighted noun topic RDM	0.9443
verb constraint error RDM	0.9120

6. Illustrative topic modelling results

Table S2 shows example topics resulting from topic modelling based on the co-occurrence between verb and DO noun, as reported in the main text. Topics were sorted in descending order of topic informativeness (see definition in SI section 5: the higher the informativeness, the more coherent the top words are in each topic), and the first 50 topics are listed in Table S2. Note that the first column (Topic ID) refers to the original index in the topic modelling results. We also calculated the mean probability of each topic by averaging the conditional probability of each topic (i.e., P(topic|verb)) across the 4,217 verbs in the training dataset. This indicates each topic's overall involvement as determined by large-scale corpora data. We found that topic mean probability is significantly correlated with topic informativeness (Pearson's $r = 0.55$, $p = 2.27 \times 10^{-17}$), suggesting that topics with higher informativeness are more often used to represent a verb's semantic constraints and play a stronger role in modelling the semantics of our stimuli.

Table S2 Top 5 words of the first 50 topics in descending order of topic informativeness

Topic ID	Top 5 words (DO nouns)					Mean probability	Informative-ness
59	animal (0.048)	bird (0.036)	horse (0.028)	dog (0.026)	cat (0.020)	0.014	0.623
80	time (0.121)	day (0.104)	year (0.055)	hour (0.055)	night (0.041)	0.008	0.619
29	shirt (0.023)	uniform (0.021)	dress (0.020)	hat (0.019)	clothes (0.019)	0.006	0.594
110	head (0.069)	hand (0.065)	arm (0.038)	leg (0.037)	finger (0.034)	0.019	0.566
22	car (0.073)	ship (0.063)	boat (0.053)	vehicle (0.040)	train (0.031)	0.011	0.562
40	member (0.033)	officer (0.019)	minister (0.018)	bishop (0.016)	representa-tive (0.016)	0.007	0.536
2	name (0.168)	word (0.145)	language (0.046)	phrase (0.034)	term (0.025)	0.008	0.524
134	child (0.159)	baby (0.053)	people (0.034)	patient (0.034)	son (0.033)	0.015	0.522
183	wine (0.041)	beer (0.041)	drink (0.035)	water (0.034)	tea (0.025)	0.007	0.512
9	relationship (0.148)	link (0.061)	friendship (0.054)	connection (0.043)	relation (0.042)	0.003	0.511
83	fire (0.162)	flame (0.057)	candle (0.047)	light (0.043)	lamp (0.026)	0.003	0.502
164	question (0.427)	call (0.030)	query (0.029)	enquiry (0.016)	prayer (0.016)	0.002	0.489
118	fish (0.032)	egg (0.031)	onion (0.020)	potato (0.017)	meat (0.013)	0.008	0.487
128	people (0.047)	man (0.032)	person (0.025)	woman (0.023)	prisoner (0.020)	0.017	0.471
19	tree (0.054)	seed (0.049)	plant (0.045)	crop (0.043)	flower (0.019)	0.008	0.469
132	protein (0.038)	gene (0.028)	acid (0.020)	molecule (0.018)	cell (0.016)	0.009	0.468

3	muscle (0.046)	heart (0.038)	skin (0.031)	body (0.031)	mind (0.027)	0.011	0.468
124	song (0.154)	music (0.029)	verse (0.028)	tune (0.026)	lyric (0.023)	0.005	0.466
15	problem (0.211)	issue (0.119)	situation (0.038)	matter (0.031)	dispute (0.026)	0.004	0.460
68	money (0.112)	fund (0.048)	amount (0.042)	resource (0.037)	sum (0.034)	0.008	0.460
64	crime (0.055)	offence (0.034)	murder (0.030)	act (0.025)	suicide (0.021)	0.005	0.458
103	disease (0.057)	infection (0.029)	virus (0.021)	condition (0.020)	problem (0.019)	0.004	0.454
48	error (0.046)	mistake (0.038)	lack (0.025)	sin (0.024)	failure (0.021)	0.005	0.452
112	right (0.225)	freedom (0.051)	interest (0.050)	liberty (0.027)	copyright (0.025)	0.003	0.451
42	friend (0.027)	woman (0.024)	man (0.021)	girl (0.019)	wife (0.017)	0.014	0.446
90	cost (0.155)	risk (0.057)	loss (0.044)	expense (0.037)	liability (0.030)	0.004	0.418
26	fee (0.059)	price (0.043)	debt (0.030)	tax (0.025)	rate (0.023)	0.006	0.413
147	interest (0.043)	feeling (0.030)	fear (0.028)	desire (0.022)	appetite (0.022)	0.008	0.413
38	area (0.043)	territory (0.041)	city (0.034)	land (0.031)	country (0.030)	0.010	0.411
144	contract (0.109)	agreement (0.086)	deal (0.070)	treaty (0.048)	term (0.014)	0.003	0.410
54	boundary (0.161)	border (0.062)	line (0.046)	limit (0.046)	distinction (0.037)	0.002	0.409
50	food (0.056)	meal (0.029)	meat (0.022)	bread (0.019)	cake (0.017)	0.009	0.405
66	road (0.053)	river (0.029)	street (0.028)	route (0.024)	lake (0.016)	0.009	0.404
47	water (0.077)	air (0.038)	gas (0.031)	blood (0.030)	oil (0.018)	0.012	0.404
104	goal (0.094)	point (0.055)	win (0.036)	victory (0.034)	score (0.026)	0.004	0.404
21	aircraft (0.065)	plane (0.051)	flag (0.048)	helicopter (0.021)	balloon (0.019)	0.004	0.402
25	way (0.089)	mile (0.051)	distance (0.037)	round (0.036)	street (0.019)	0.014	0.392
88	room (0.077)	house (0.037)	building (0.027)	space (0.021)	floor (0.019)	0.011	0.392
32	concern (0.040)	opinion (0.035)	view (0.027)	feeling (0.024)	interest (0.020)	0.005	0.391
6	hill (0.040)	mountain (0.034)	wall (0.030)	slope (0.025)	step (0.023)	0.007	0.386
13	hole (0.119)	grave (0.028)	well (0.024)	pit (0.022)	trench (0.021)	0.004	0.386
45	law (0.094)	rule (0.055)	order (0.031)	regulation (0.026)	decision (0.022)	0.007	0.385
158	role (0.043)	duty (0.041)	dream (0.035)	objective (0.031)	aim (0.031)	0.003	0.380

182	building (0.050)	house (0.037)	church (0.022)	bridge (0.021)	tower (0.016)	0.006	0.377
171	staff (0.032)	worker (0.020)	officer (0.018)	employee (0.014)	member (0.013)	0.006	0.375
169	force (0.064)	army (0.051)	troop (0.034)	ship (0.024)	enemy (0.021)	0.009	0.372
121	people (0.045)	audience (0.041)	reader (0.025)	fan (0.019)	crowd (0.016)	0.024	0.367
176	meeting (0.055)	event (0.042)	conference (0.031)	session (0.020)	workshop (0.017)	0.004	0.365
151	authority (0.021)	company (0.020)	doctor (0.012)	police (0.012)	employer (0.011)	0.008	0.363
1	team (0.111)	winner (0.045)	player (0.044)	candidate (0.029)	side (0.028)	0.006	0.362

References

1. Elman JL (2009) On the meaning of words and dinosaur bones: Lexical knowledge without a lexicon. *Cognitive science* 33(4):547-582.
2. McRae K, Hare M, Elman JL, & Ferretti T (2005) A basis for generating expectancies for verbs from nouns. *Memory & Cognition* 33(7):1174-1184.
3. Bicknell K, Elman JL, Hare M, McRae K, & Kutas M (2010) Effects of event knowledge in processing verbal arguments. *Journal of memory and language* 63(4):489-505.
4. Klimovich-Gray A*, et al.* (2019) Balancing Prediction and Sensory Input in Speech Comprehension: The Spatiotemporal Dynamics of Word Recognition in Context. *Journal of Neuroscience* 39(3):519-527.
5. Kocagoncu E, Clarke A, Devereux BJ, & Tyler LK (2017) Decoding the cortical dynamics of sound-meaning mapping. *Journal of Neuroscience* 37(5):1312-1319.
6. Mimno D, Wallach HM, Talley E, Leenders M, & McCallum A (2011) Optimizing semantic coherence in topic models. *Proceedings of the conference on empirical methods in natural language processing*, (Association for Computational Linguistics), pp 262-272.
7. Newman D, Lau JH, Grieser K, & Baldwin T (2010) Automatic evaluation of topic coherence. *Human Language Technologies: The 2010 Annual Conference of the North American Chapter of the Association for Computational Linguistics*, (Association for Computational Linguistics), pp 100-108.
